# A Virtual Companionship Intervention Reduces Loneliness During the COVID-19 Pandemic

**DOI:** 10.1093/geroni/igab046.3451

**Published:** 2021-12-17

**Authors:** Ellen Rudy, Kelsey McNamara, Rajiv Patel, Corey Sturm

**Affiliations:** Papa Inc., Miami, Florida, United States

## Abstract

Loneliness and social isolation are established risk factors for many clinical conditions yet few scalable interventions exist. Papa Inc. is a national service that pairs older adults with “Papa Pals” (empathetic, laypeople) who provide companionship and assistance with everyday tasks. Participants have free access if their Medicare Advantage plan offers it. During the COVID-19 pandemic, Papa provided virtual companionship visits via telephone or video. This study evaluated the impact of virtual companionship visits on loneliness status (UCLA 3-item Loneliness Scale) during the COVID-19 pandemic. The sample (N=894) included adults ages 65+ who identified as lonely at baseline and who completed at least one virtual visit between March 18, 2020 and December 31, 2020. Virtual visits were classified into four categories based on participants’ total number of visit minutes: Low (124 ave min), Medium Low (ML) (305 ave min), Medium High (MH) (567 ave min), and High (1360 ave min). Lonely and severely lonely participants engaged a mean of 573 and 673 minutes in the program, respectively. Improvement in loneliness status was associated with greater use of minutes for the ML and MH participants compared to Low participants (ML OR: 1.46 95CI: 1.00 - 2.11, MH OR 1.65 95CI: 1.13 - 2.40). These findings indicate that a virtual companionship intervention can be an impactful and scalable tool for older adults who want to age at home and have limited social support, especially during the uncertain COVID landscape. Further research is warranted to understand persistent loneliness.

